# Supplementary material for: Lon1 Inactivation Downregulates Autophagic Flux and Brassinosteroid Biogenesis, Modulating Mitochondrial Proportion and Seed Development in Arabidopsis
Source: Int J Mol Sci. 2024 May 16;25(10):5425. doi: 10.3390/ijms25105425 (PMC11121791; doi:10.3390/ijms25105425)
Supplement: Supplementary file 1 [file ijms-25-05425-s001.zip › figure-S1.pdf]

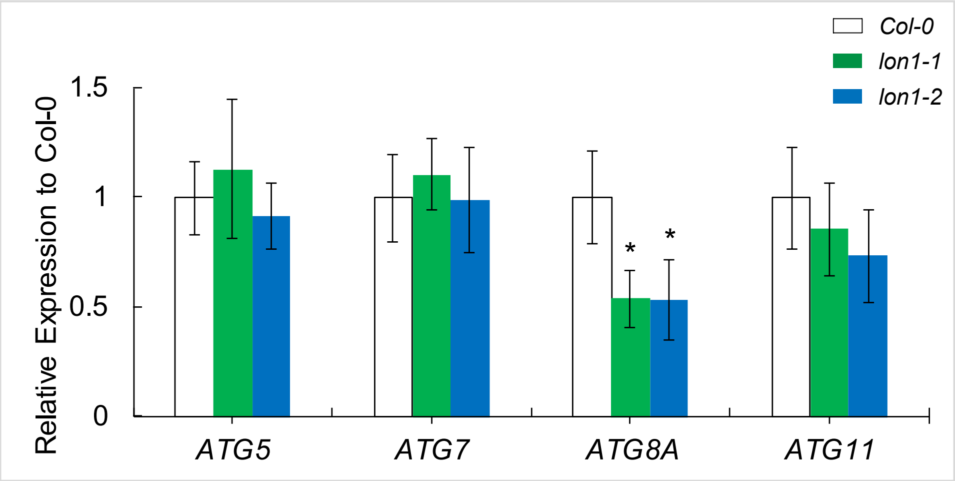

Fig S1. RT-PCR validation of RNAseq data autophagy core genes

Quantitative real-time PCR (qRT-PCR) was conducted to assess the transcript levels of selected autophagy core genes (*ATG5*, *ATG7*, *ATG8A*, *ATG11*) in ten-day-old *Col-0*, *lon1-1*, and *lon1-2* mutant seedlings. Error bars represent standard deviations of four biological replicates. Statistical significance was determined using Student’s T-test (* indicates $P<0.05$). This supports Fig 1.
