# Supplementary material for: Lon1 Inactivation Downregulates Autophagic Flux and Brassinosteroid Biogenesis, Modulating Mitochondrial Proportion and Seed Development in Arabidopsis
Source: Int J Mol Sci. 2024 May 16;25(10):5425. doi: 10.3390/ijms25105425 (PMC11121791; doi:10.3390/ijms25105425)
Supplement: Supplementary file 1 [file ijms-25-05425-s001.zip › figure-S2.pdf]

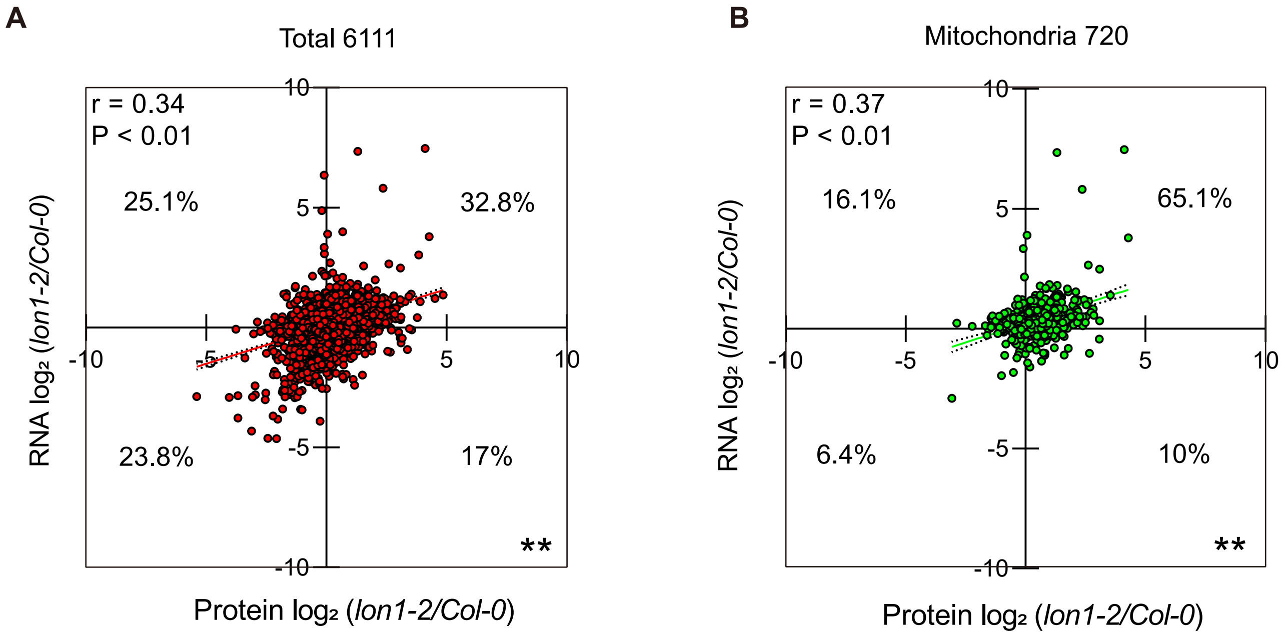

Figure S2. Correlation graphs between transcriptomic and proteomic data in *lon1-2* compared with *Col-0*.

(A) Dot plots visualizing log-transformed data of 6,111 genes for transcriptomic versus proteomic comparison in *lon1-2* mutant line compared with *Col-0*. (B) Dot plots visualizing log-transformed data of 720 mitochondrial genes for transcriptomic versus proteomic comparison in *lon1-2*. The numbers in the top left corner indicate the Pearson correlation coefficients. Statistical significance is denoted by ** for $P < 0.01$.
