# Supplementary material for: Lon1 Inactivation Downregulates Autophagic Flux and Brassinosteroid Biogenesis, Modulating Mitochondrial Proportion and Seed Development in Arabidopsis
Source: Int J Mol Sci. 2024 May 16;25(10):5425. doi: 10.3390/ijms25105425 (PMC11121791; doi:10.3390/ijms25105425)
Supplement: Supplementary file 1 [file ijms-25-05425-s001.zip › figure-S3.pdf]

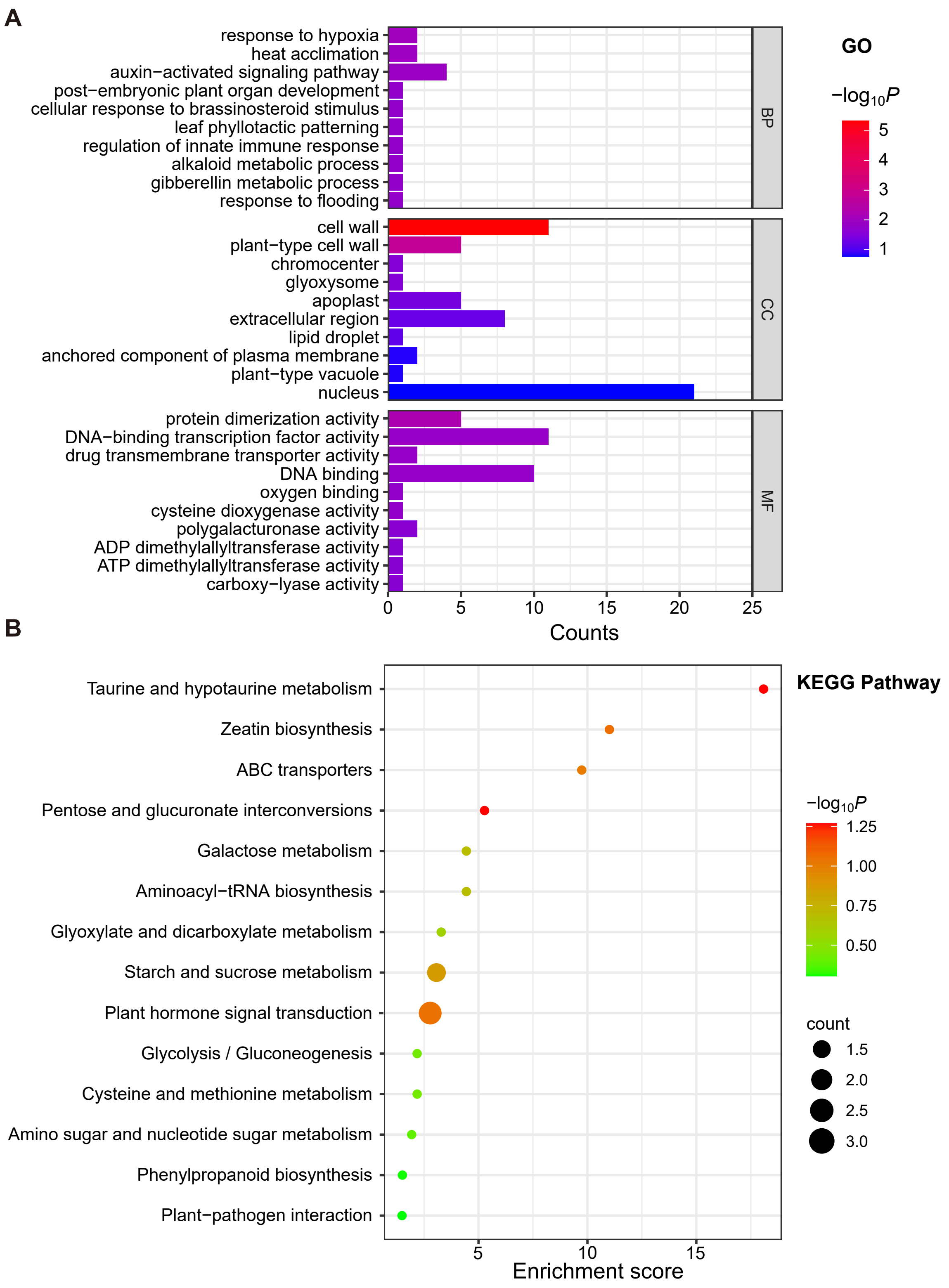

Figure S3 Commonly up-regulated genes in *lon1-2*, *atg5-1* and *lon1-2atg5-1* are enriched with cell wall processes.

(A) A total of 92 genes were commonly upregulated in *lon1-2*, *atg5-1*, and *lon1-2atg5-1*, and were subjected to Enrichment of Gene Ontology (GO). The horizontal bars represent the counts for Biological Processes (BP), Cell Compartments (CC) and Molecular Functions (MF), while the blue-red color bar indicates the range of Log-transformed P values. (B) KEGG pathway enrichment analysis of down-regulated genes in all three mutants. The size of the bubbles corresponds to the number of genes involved, while the green-red color bar indicates the range of Log-transformed P values.
