# Supplementary material for: Lon1 Inactivation Downregulates Autophagic Flux and Brassinosteroid Biogenesis, Modulating Mitochondrial Proportion and Seed Development in Arabidopsis
Source: Int J Mol Sci. 2024 May 16;25(10):5425. doi: 10.3390/ijms25105425 (PMC11121791; doi:10.3390/ijms25105425)
Supplement: Supplementary file 1 [file ijms-25-05425-s001.zip › figure-S5.pdf]

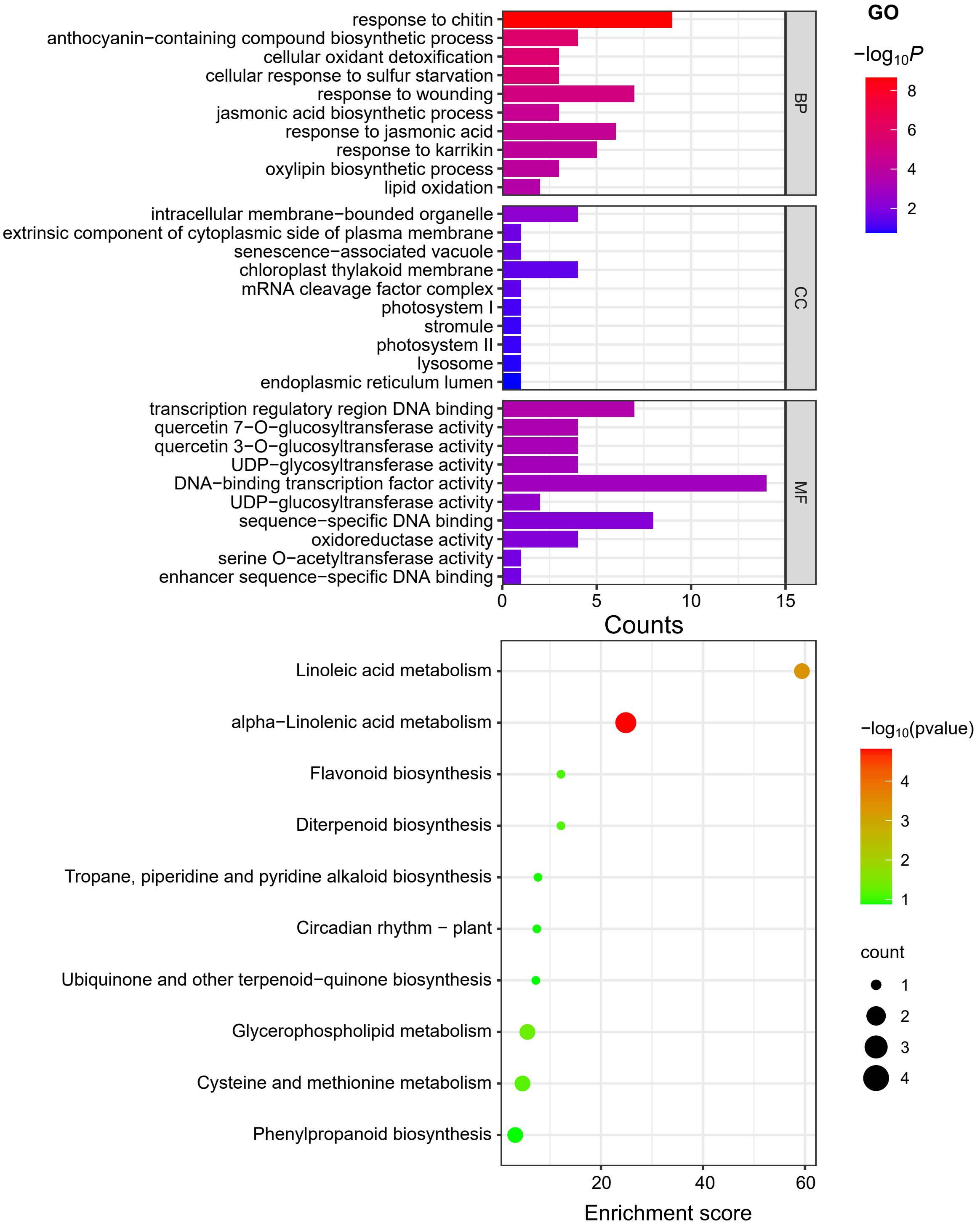

Figure S5 Genes which were down-regulated in *lon1-2* but up-regulated in *atg5-1* are enriched with response to chitin pathway.
(A) A total of 86 genes were upregulated in *lon1-2* but downregulated in *atg5-1*, and were subjected to Enrichment of Gene Ontology (GO) analysis. The horizontal bars represent the counts for Biological Processes (BP), Cell Compartments (CC) and Molecular Functions (MF), while the blue-red color bar indicates the range of Log-transformed P values. (B) KEGG pathway enrichment analysis of down-regulated genes in all three mutants. The size of the bubbles corresponds to the number of genes involved, while the green-red color bar indicates the range of Log-transformed P values.
